# Gene Expression Changes Related to Endocrine Function and Decline in Reproduction in Fathead Minnow (*Pimephales promelas*) after Dietary Methylmercury Exposure

**DOI:** 10.1289/ehp.8786

**Published:** 2006-05-30

**Authors:** Rebecca Klaper, Christopher B. Rees, Paul Drevnick, Daniel Weber, Mark Sandheinrich, Michael J. Carvan

**Affiliations:** 1 Great Lakes WATER Institute, University of Wisconsin–Milwaukee, Milwaukee, Wisconsin, USA; 2 Department of Zoology, Miami University, Oxford, Ohio, USA; 3 Marine and Freshwater Biomedical Sciences Center, University of Wisconsin–Milwaukee, Milwaukee, Wisconsin, USA; 4 River Studies Center and Department of Biology, University of Wisconsin–La Crosse, La Crosse, Wisconsin, USA

**Keywords:** endocrine disruption, gene expression, mercury, methylmercury, microarray, *Pimephales promelas*, reproduction, toxicogenomics, vitellogenin

## Abstract

**Background:**

Methylmercury (MeHg) is a known neurotoxic agent, but the mechanisms by which MeHg may act on reproductive pathways are relatively unknown. Several studies have indicated potential changes in hormone levels as well as declines in vertebrates with increasing dietary MeHg exposure.

**Objectives:**

The purpose of this study was to identify alterations in gene expression associated with MeHg exposure, specifically those associated with previously observed changes in reproduction and reproductive biomarkers. Fathead minnows, *Pimephales promelas*, were fed one of three diets that were similar to documented concentrations of MeHg in the diets of wild invertivorous and piscivorous fish. We used a commercial macroarray in conjunction with quantitative polymerase chain reaction to examine gene expression in fish in relation to exposure to these environmentally relevant doses of MeHg.

**Results:**

Expression of genes commonly associated with endocrine disruption was altered with Hg exposure. Specifically, we observed a marked up-regulation in vitellogenin mRNA in individual Hg-exposed males and a significant decline in vitellogenin gene expression in female fish with increasing Hg concentrations. Other genes identified by the macroarray experiment included those associated with egg fertilization and development, sugar metabolism, apoptosis, and electron transport. We also observed differences in expression patterns between male and female fish not related to genes specifically associated with reproduction, indicating a potential physiological difference in the reaction of males and females to MeHg.

**Conclusion:**

Gene expression data may provide insight into the mechanisms by which MeHg affects reproduction in fish and indicate how MeHg differs in its effect from other heavy metals and endocrine-disrupting compounds.

Mercury is prevalent in the environment as a result of both natural processes and emissions from anthropogenic sources (Keating et al. 1997; Wiener et al. 2003). However, atmospheric deposition from anthropogenic emissions such as coal power plants is frequently the major source of Hg in aquatic systems (Landis and Keeler 2002). After deposition, inorganic Hg is methylated by microbes, then biomagnified in aquatic food webs. Subsequently, the greatest concentrations of methylmercury (MeHg) are found in piscivorous fish and wildlife (Spry and Wiener 1991; Watras and Bloom 1992). MeHg is the most toxic form of Hg, and nearly all (95–99%) Hg in fish is MeHg (Bloom 1992; Grieb et al. 1990).

The environmental risks associated with MeHg have been explored mainly in relationship to the risks to human health associated with consumption of contaminated fish. The main route of MeHg exposure in humans is through fish consumption, and the body burden of MeHg in humans is directly correlated to the quantities of fish consumed (Bjornberg et al. 2005; Cole et al. 2004). Hg in its methylated form is neurotoxic, particularly to developing nervous systems, and has been associated with many different neurological problems, from learning disabilities and behavioral changes to death (National Research Council 2000; Zelikoff et al. 1995). However, the mechanistic effects of MeHg on other physiological processes such as reproduction are relatively unknown, and there are comparatively few studies that examine risks of MeHg exposure on fish populations themselves (Armstrong 1979; Spry and Wiener 1991; Wiener et al. 2003).

The few studies on the impact of MeHg contamination on fish have shown consequences for reproduction. For example, fat-head minnows (FHM), *Pimephales promelas*, fed MeHg-contaminated diets that simulated concentrations found in zooplankton, invertebrates, and small fish in North America showed a delay in spawning, a decline in spawning activity, and a decline in the number of eggs laid (Hammerschmidt et al. 2002) with increasing MeHg. Dietary MeHg impairs gonadal development in walleye (*Sander vitreus*; Friedmann et al. 1996), FHM (Hammerschmidt et al. 2002) and walking catfish (*Clarias batrachus*; Kirubagaran and Joy 1988, 1992) and causes testicular atrophy in guppies (Wester 1991).

The mechanism by which MeHg alters reproduction is unclear. However, there is some indication that MeHg suppresses sex hormones that elicit secondary sex characteristics and stimulate gonadal development and game-togenesis. In the companion study (Drevnick and Sandheinrich 2003) to the present article, exposure to environmentally relevant concentrations of MeHg inhibited gonadal development and suppressed estrogen production in female FHM and testosterone in male FHM. Male fish fed a control diet had mean testosterone concentrations that were 20 and 116% greater than those fed low-MeHg (0.87 μg/g dry wt) and medium-MeHg (3.93 μg/g dry wt) diets, respectively. Female fish fed a control diet had mean estrogen concentrations that were 164 and 416% greater than those fed low- and medium-MeHg diets. Other studies have found similar results in fish at various concentrations (Arnold et al. 1998; Fynn-Aikins et al. 1998). MeHg also interferes with vitellogenesis (Kirubagaran and Joy 1988) and spermatogenesis (Kirubagaran and Joy 1992).

Genomic markers provide a useful tool to examine the physiological mechanisms that may be affected by toxicant exposure [for review see Klaper and Thomas (2004)]. Genomic techniques allow simultaneous measurement of multiple biochemical pathways at levels of sensitivity not available with other biomarkers. Moreover, they provide information that cannot be obtained with currently available tests such as traditional ELISA assays (Klaper and Thomas 2004).

Recent evidence has demonstrated the utility of genomic techniques in assessing the effects of dietary MeHg on fish. Using real-time quantitative polymerase chain reaction (qPCR), Gonzalez et al. (2005) examined the expression of 13 genes in muscle, liver, and brain tissue of zebrafish (*Danio rerio*). Dietary exposures of 5 and 13.5 μg MeHg/g dry wt increased expression of genes cytochrome *c* oxidase subunit I (*coxI*) and cytoplasmic superoxide dismutase (*sod*) associated with mitochondrial metabolism and production of reactive oxygen species (ROS) in liver and skeletal muscle. The specific changes in gene expression in that study provide an indication of the potential mechanisms by which MeHg may be affecting these tissues. However, the study did not examine gene expression in relation to a specific physiological response and did not examine genes involved in reproduction pathways, and exposures were higher than normal environmental exposures.

In the research presented here we assessed gene expression in FHM fed diets contaminated with environmentally relevant concentrations of MeHg as a continuation of the previous study by Drevnick and Sandheinrich (2003) that found altered reproduction associated with increased MeHg exposure. We used a combination of genomic techniques, including a commercial macroarray, a suppressive subtraction hybridization, and qPCR, to identify differentially expressed genes associated with MeHg exposure in the FHM. The goal of this research was to begin to examine the mechanistic relationship between reproductive changes and gene expression and to identify potential endocrine changes that may be linked to MeHg exposure.

## Materials and Methods

### Test organisms

We obtained embryos of FHM from the Upper Midwest Environmental Sciences Center (U.S. Geological Survey, La Crosse, WI) and raised them to maturity. Before exposure, larvae were fed *Artemia nauplii* for 60 days and then fed Sterling Silver Cup Fish Food (Nelson and Sons Inc., Murray, UT) for 30 days. Subsequently, 200 fish were transferred to each of fifteen 180-L flow-through aquaria for exposure studies. Aquaria were filled with well water, temperature was maintained at 23.6 ± 0.1°C, and each received a 16/8-hr light/dark photoperiod cycle. Additional information on the experimental design and culturing of FHM can be found in Drevnick and Sandheinrich (2003).

### MeHg exposure treatments and RNA extraction

Minnows were fed food containing one of three concentrations of MeHg at 5% body mass per day: control (0.058 ± 0.004 μg/g dry wt), low MeHg (0.87 ± 0.02 μg/g dry wt), or medium MeHg (3.93 ± 0.08 μg/g dry wt). All three concentrations were designed to represent the diets of insectivorous and piscivorous fish from some midcontinental low-alkalinity lakes contaminated with Hg from non-point sources (Hammerschmidt et al. 2002). Diets were prepared by mixing food with methyl-mercuric chloride in alcohol. Control diets were mixed with alcohol alone. Alcohol was evaporated from the food, and the diet was frozen until use. Fish were fed their respective food treatment for 600 days. Fish in this study were used in accordance with protocols approved by the University of Wisconsin–La Crosse Institutional Animal Care and Use Committee. Animals used were treated humanely and with regard for the alleviation of suffering. After 600 days the fish were euthanized. The liver was harvested, placed in 2 mL of Trizol reagent (Invitrogen, Carlsbad, CA) and kept at –80°C until extraction. RNA was extracted per manufacturer’s intructions for Trizol.

### Macroarray construction and analysis

The macroarray used in the experiment was a commercially available 200-gene FHMinnow array from EcoArray, Inc. (Alachua, FL; http://www.ecoarray.com/). Arrays were constructed, hybridized, and analyzed at EcoArray as previously described (Larkin et al. 2002, 2003). Arrays consisted of cDNA spotted in duplicate onto 11.5 × 7.6-cm neutral nylon membranes. Controls included water blanks, Cot-1 repetitive sequences, M13 sequence, and exogenous spiking genes from *Arabidopsis thaliana* used for normalization purposes (SpotReport 3; Stratagene, La Jolla, CA). Total RNA was DNase treated (Ambion, Austin, TX) and radiolabeled with (α-^33^P)dATP (Strip-EZ RT; Ambion), and 0.6 ng of spiking RNA was added to each labeling reaction. After hybridization, we quantitatively evaluated the arrays using a Typhoon 8600 imaging system (Amersham Pharmacia Biotech, Molecular Dynamics, Sunnyvale, CA).

### Macroarray experimental design

We exposed sixteen membranes for the macroarray experiment. Four fish of each sex were chosen from both the control and highest MeHg dose treatment based on highest RNA quality as determined by absorbance at 260/280 nm and gel electrophoresis. Each sample was separately exposed to array. For each cDNA clone represented on the membrane, we substracted the general background of each membrane from the average value of the duplicate spots on the membrane. The values were then normalized to the average value of two spiking genes that were spotted on the membranes. Data from control and MeHg-treated fish were compared with a *t*-test. Additional information about genes found on the 200-gene FHMinnow array is provided in the Supplemental Material available online for this article (http://www.ehponline.org/members/2006/8786/supplemental.pdf).

### qPCR analysis

qPCR was performed for 10 individuals from each treatment for a select number of genes. Genes were selected by cross-referencing differentially expressed genes in the macroarray data with genes found in a suppressive subtraction hybridization. Because gene sequence is necessary to design primers for qPCR, genes identified through suppressive subtractions that also showed significant differential expression on macroarrays were chosen for qPCR analysis. These included FHM vitellogenin [*vtg*, GenBank accession no. AF130354; http://www.ncbi.nih.gov)] and zona pellucida 2 (*zp2*, GenBank accession no. EB684274). In addition apolipoprotein (*apo*, GenBank accession no. EB684272) and nuclear autoantigenic sperm protein (*nasp*, GenBank accession no. EB684273 ) were also identified through suppressive subtraction and were chosen because of their role in reproduction.

Primers for PCR were selected in areas of intron/exon junctions (where possible) to minimize the potential for DNA amplification during qPCR. To determine splice sites, each gene sequence was aligned to zebrafish sequences [ZFIN database (Zebrafish Information Network; http://zfin.org)] using bl2seq (Tatusova and Madden 1999] and compared to intron–exon junctions on the ZFIN database. Primers (Table 1) were chosen to amplify approximately 150 bp of each target gene and designed to have approximately similar melting temperatures (52–58°C) and a guanine and cytosine content of 40–60% with minimal repeats. All primers were synthesized and HPLC purified at Integrated DNA Technologies (Coralville, IA).

**Figure 1:**
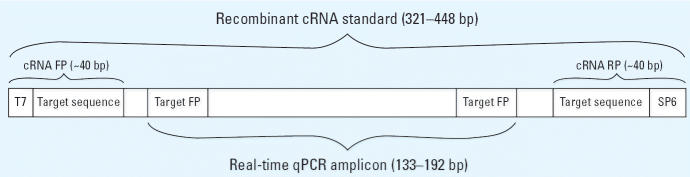
Diagram demonstrating design of cRNA standards for qPCR.

Recombinant cRNA standards were used for quantification using standard curves as described (Rees and Li 2004; Vanden Heuvel 1998). Briefly, cRNA standards for each of the genes listed above ranged in size from 321 to 448 bp (Table 1). A cRNA PCR product containing a 5′ T7 promoter, a region of targetgene–specific sequence including the region of the real-time amplicon, and an SP6 promoter at the 3′ end (Figure 1) was created using cDNA from a sample (GeneAmp RNA PCR Kit; Applied Biosystems, Foster City, CA). The cRNA product was reamplified, cleaned (QIAquick PCR Purification Kit; Qiagen, Valencia, CA), and transcribed using the Riboprobe In Vitro Transcription System (Promega Corp., Madison, WI) according to standard protocol. Each cRNA was DNase-treated (Promega Corp.) with RQ1 RNase-free DNase followed by phenol:chloroform extraction (24:1). The aqueous phase was isolated and extracted with chloroform–isoamyl alcohol (24:1) followed by an overnight ethanol precipitation at –20°C. Each cRNA pellet was resuspended in 20 μL RNase-free water (Gibco, Invitrogen, Grand Island, NY) and filtered through a NucAway Spin Column (Ambion) to remove free nucleotides. The size and quality of the cRNA standard were verified by analysis on an agarose gel and quantified at 260 nm.

First-strand cDNA for samples was synthesized using the GeneAmp RNA PCR kit (Ambion). Reactions were incubated at room temperature for 10 min; incubated for 30 min at 42°C, 5 min at 99°C; then stored at –20°C until used for template in PCR. Products were quantified using Brilliant SYBR Green QPCR (Stratagene) according to manufacturer’s directions and 300 nM of each primer. PCR reactions were analyzed on an MJ Research DNA Engine Opticon System (BioRad Laboratories, Waltham, MA) under the following conditions: 1 cycle of 95°C for 5 min, 40 cycles of 94°C for 30 sec, 56°C for 30 sec, 72°C for 1 min, and 1 cycle at 72°C for 10 min. The cycle threshold [C(t)] was manually set at 0.005 for all plates. A melting curve was generated to check for presence of nonspecific PCR products. Target gene samples were performed in triplicate, and copy number was estimated against a standard curve. Negative PCR controls were used to check for genomic DNA contamination, and all real time-reverse transcriptase PCR products were run on agarose gels. Treatments were compared using a Kruskall-Wallis test as analyzed with SPSS (version 13.0; SPSS, Inc., Chicago, IL).

## Results

### Detection of changes in gene expression using macroarray

Dietary MeHg altered the expression of dozens of genes in liver tissue, including those related to reproduction. Using the EcoArray macroarray, we were able to identify 76 genes in female FHM and 42 in males that were differentially expressed (2-fold difference) between the highest-MeHg-exposed fish and controls as determined by comparing the average for each sex over each treatment. However, large variation in response among individual fish and small sample size (*n* = 4 macroarrays per sex, per treatment) resulted in a large number of statistically nonsignificant values (Tables 2, 3).

**Figure 2:**
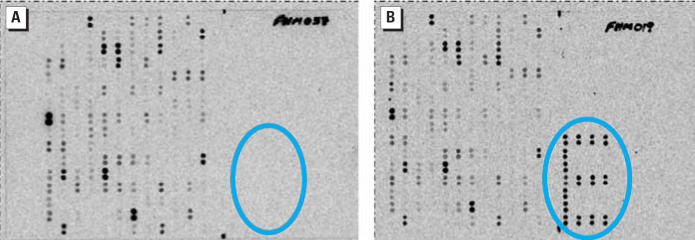
EcoArray macroarray of 200 genes exposed to cDNA from (*A*) a single control male fish and (*B*) a fish exposed to a diet of 4 μg MeHg/g dry wt. Blue circles show location on the array where vitellogenin was spotted in duplicate in the shape of an “E.” Vitellogenin mRNA was expressed to a greater extent in the exposed fish (*B*) where the spots are clearly visible than in the control (*A*) where no spots are visible for vitellogenin.

Gene expression differed by sex, and the effects of treatment on gene expression differed by sex. Vitellogenin mRNA, a phosphoglycolipoprotein associated with egg production and normally produced solely in female fish, increased an average 142.8-fold over controls in male fish. Macroarrays were spotted with multiples of this gene because it is of interest in endocrine disruption studies (circled in Figure 2B). Control male fish (Figure 2A) did not express vitellogenin RNA, although several treated males had large amounts of vitellogenin RNA (Figure 2B). This result was highly variable among treated males. In females, vitellogenin mRNA expression was down-regulated 0.6× on average in treated fish.

Expression of RNA coding for zona pellucida proteins ZP2 and ZP3 also appeared to be up-regulated in individual male (2.8× and 14.7×, respectively) and female fish (9.5× and 11.1×, respectively), although the increase was statistically nonsignificant. Genes that were significantly down-regulated and statistically significant in males included phosphoethanolamine methyltransferase (0.2× change treated vs. control) and glucose 6-phosphatase (0.4×). In males, retinal binding protein 4 increased 2-fold (*p* = 0.0159) and 4-hydroxyphenylpyruvate dioxygenase and α-amylase were also up-regulated (1.9×), but the statistical significance was slightly > *p* = 0.05 (*p* = 0.0664 and *p* = 0.0895, respectively) (Table 3). Genes putatively up-regulated in females included glyceraldehyde 3-phosphate dehydrogenase (*gapdh*) (1.7×, *p* = 0.0263). Interestingly, *gapdh* is a common cellular component and is considered to be expressed constitutively; therefore it is often used as a gene for normalization of qPCR data. It was not used here because of its change with exposure.

### qPCR analysis of differential gene expression

**Figure 3:**
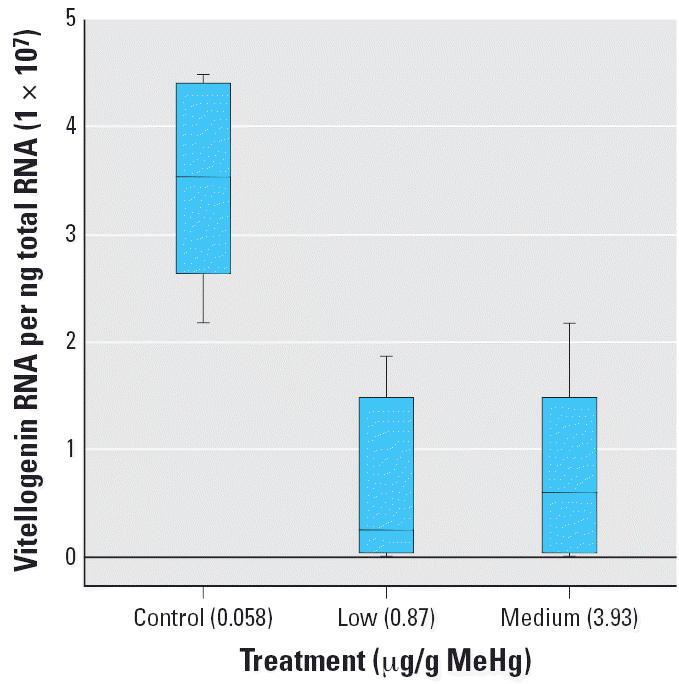
Box plot of molecules of vitellogenin RNA per nanogram of total RNA for female FHM treated with three different MeHg-contaminated diets at 5% body mass per day (10 fish per treatment); control, 0.058 ± 0.004 μg MeHg/g dry wt; low, 0.87 ± 0.02 μg MeHg/g dry wt; medium, 3.93 ± 0.08 μg MeHg/g dry wt. Data indicate suppressed vitellogenin RNA expression with increasing MeHg exposure (χ^2^ = 10.835, *df* = 2, *p* = 0.004). Boxes represent the interquartile range (first to third quartiles of data) with the middle lines indicating the median and bars indicating the range of data.

qPCR supported the results of macroarray experiments for vitellogenin, apolipoprotein, and *nasp*, with differences only in the scale of estimated up-regulation or down-regulation and the significance of the effect of treatment; macroarrays underestimated effects compared with qPCR results. MeHg-treated female fish showed significantly lower expression in vitellogenin RNA than in control fish (*p* = 0.004, χ^2^ = 10.835, *df* = 2; Figure 3). Both MeHg treatments down-regulated vitellogenin RNA production to almost the same magnitude. Using the macroarray, vitellogenin was down-regulated approximately 2-fold; however, qPCR estimated a 4-fold down-regulation in female fish. The significance of changes in female vitellogenin mRNA expression also increased with qPCR measurements either due to technique or to the inclusion of all of the individuals from the experiment (*n* = 10 vs. *n* = 4). From qPCR results, it appears that genes approaching 1.7× over controls may also be differentially regulated.

Dietary MeHg up-regulated expression of the vitellogenin gene in livers of several of the male fish in both MeHg treatments; none of the male fish fed control diets expressed the gene. However, there was significant variability in response in MeHg-fed males, with some of the treated fish showing no up-regulation and some with significant up-regulation (ranging from 0 to 200× over control males). This contributed to the overall average increase in expression and also to the lack of statistical significance (Table 4). This was similar to the findings of the macroarrays, where vitellogenin RNA was up-regulated 142× with variation among individuals leading to insignificant statistical measures across the group as a whole.

Dietary MeHg also altered expression of apolipoprotein in females, causing an increase in expression of approximately 4-fold in both treatments (χ^2^ = 8.861, *df* = 2, *p* = 0.012) and increased *nasp* by 20–40× (χ^2^ = 6.151, *df* = 2, *p* = 0.046). Zona pellucida expression, also commonly measured in association with endocrine disruption, did not appear to vary with exposure.

## Discussion

In this study, we assessed gene expression in FHM fed diets with up to 4 μg MeHg/g dry wt—environmentally relevant concentrations that have been shown previously to suppress the production of sex hormones and alter reproduction in minnows (Drevnick and Sandheinrich 2003; Hammerschmidt et al. 2002). Many industrial chemicals have been linked to the disruption of endocrine function (Colborn et al. 1993), but research has focused primarily on compounds that are similar in structure to estrogens. It is possible that MeHg may act as an endocrine disruptor by binding to estrogen receptors and acting essentially as an estrogen mimic. Cadmium, cobalt, nickel, lead, and Hg have been shown to activate estrogen receptors and increase their production (Garcia-Morales et al. 2004; Henson and Chedrese 2004; Johnson et al. 2003; Martin et al. 2003; Stoica et al. 2000). However, there are notable differences between genes differentially expressed in this study and those identified in studies of other metals. Our data indicate that MeHg may have a different mode of action than other heavy metals, particularly at environmentally relevant exposure levels for MeHg. Studies have indicated that heavy metals may affect reproduction through interference with estrogen-responsive elements (Vetillard and Bailhache 2005) but invariably cause changes in ROS, which may also be the basis for changes in endocrine function with other Hg species and other heavy metals (Henson and Chedrese 2004; Martin et al. 2003). This is seen with cadmium, for example, which affects vitellogenin protein production but also induces *sod* and glutathione *S*-transferase (*gst*) (Olsson et al. 1995; Pereira et al. 1993; Sheader et al. 2006). We did not find significant genomic changes associated with ROS (e.g., changes in the expression of *sod*, *gst*) with MeHg exposure (these genes were on the macroarray) at these levels of MeHg. Instead, our array data indicated differential expression associated with apoptosis, phospholipid biosynthesis, sugar metabolism, interference with gonadotropin pathways, calcium regulation, phospholipid biosynthesis, and plasma transport of proteins. This may be a factor of dose, as Gonzalez et al. (2005) did find evidence of differential expression of ROS-related genes at much higher MeHg concentrations. Regardless, according to our data, there is a clear separation between the effects of oxidative stress and reproductive effects.

Differential gene expression in this study does overlap with gene expression patterns associated with known estrogenic compounds (e.g., Larkin et al. 2003), which lends support to the hypothesis of MeHg as a nonsteroidal endocrine disruptor. Of the genes that differed among treatments, vitellogenin is of particular interest due to the recommendation of assessment of vitellogenin in protocols for screening potential endocrine-disrupting chemicals (Endocrine Disruptor Screening and Testing Advisory Committee 1998; Marin and Motozzo 2004). This is due to its sensitivity as a biomarker and the correlation with adverse effects in male and female fish (e.g., Panter et al. 1998). Vitellogenin is a female-specific glucolipoprotein yolk precursor produced by all oviparous animals and is expressed specifically in the liver and transported to the gonad in females during egg development. It is up-regulated before yolk deposition and is important in egg production. Although not normally produced by male fish, its expression can be induced in males by injection of 17β-estradiol or exposure to chemicals that mimic estrogens (Byrne et al. 1989; Tong et al. 2004). When produced in males, vitellogenin is not readily removed from the bloodstream and can ultimately cause kidney and liver damage (Elliot et al. 1979; Herman and Kincaid 1988).

The presence of vitellogenin in some male fish in this experiment provides an indication that MeHg contamination may be causing an estrogenic-like effect; however, results for individual male fish were variable. Variable vitellogenin responses of males has also been found in other studies in which fish have been exposed to known endocrine-disrupting compounds in the field or laboratory (George et al. 2004; Sole et al. 2002). In each of these cases, of the total population sampled, up to 50% of fish express vitellogenin. Vitellogenin in males in the present study never reached the levels found in female fish.

In contrast, dietary MeHg significantly suppressed vitellogenin gene expression in sexually mature female fish (Figure 3), potentially affecting egg production. This is the first study to measure differences in the gene expression of vitellogenin and other genes associated with reproduction in response to environmentally realistic concentrations of MeHg. This was also associated with a decline in estrogen as well as a decline in reproduction (Drevnick and Sandheinrich 2003). In another study, Kirubagaran and Joy (1995) found that MeHg exposure decreased phospholipid content in ovarian tissue of fish and hypothesized that this is due to inhibition of vitellogenin synthesis in the liver. Vitellogenin gene expression declined in liver samples of fish exposed to MeHg, which supports this hypothesis.

Additional genomic biomarkers associated with endocrine disruption or reproduction that changed with MeHg exposure include genes responsible for proteins for egg fertilization and implantation (*zp2* and *zp3*), cholesterol and lipid transport (*apo*), and cell division (*nasp*). Expression of *apo* and *nasp* was found to increase in females that had been exposed to MeHg.

Apoptosis, variance in sugar metabolism, and calcium regulation also appear linked to MeHg exposure in this study. The enzyme GAPDH is involved in glycolysis and is proposed to play an important role in apoptosis (Chuang et al. 2005). Hypoxia and diabetes increase the expression of this enzyme in various tissues (Beisswenger et al. 2003; Graven and Farber 1995; Wentzel et al. 2003), and it was up-regulated in female fish exposed to MeHg. This may indicate a link between changes in sugar metabolism and apoptosis in liver tissue after MeHg exposure. However, this change appeared to be female specific, potentially indicating sex-specific effects of MeHg on metabolism. Other genes associated with sugar metabolism included glucose 6-phosphatase, involved in the production of glucose, and α-amylase, an enzyme that converts starches to sugars. Genes for 4-hydroxyphenylpyruvate dioxygenase, α-amylase, and retinal binding protein were all up-regulated in male fish, whereas glucose 6-phosphatase was down-regulated with MeHg exposure. Because these genes are associated with sugar metabolism, insulin function, and diabetes in mammals, our data indicate that MeHg may disrupt these functions particularly in males, and there may be a link between MeHg exposure and certain metabolic diseases.

MeHg has been shown to affect bone cells, inducing hypercalcemia in goldfish and disrupting calcium homeostasis (Suzuki et al. 2004). Calcium levels in the blood are related to estrogen levels and affect the transport and metabolism of proteins. Because many of the differentially expressed genes identified in the macroarray analysis are associated with calcium, direct disruption of calcium transport or alteration in the levels of calcium may be responsible for some of these effects.

This study suggests that MeHg acts as an endocrine disruptor and affects not only gene expression associated with reproduction and endocrine function but other pathways as well. The genomic response to MeHg appears different than the response of vertebrates to other heavy metals. As more genomic data become available for other heavy metals and endocrine-disrupting compounds, continuation of this work to evaluate unique gene expression patterns will provide specific biomarkers that indicate the impact of MeHg versus that of other toxic compounds.

## Figures and Tables

**Table 1:** Primers used for qPCR and RNA standards for qPCR.

Gene locus^a^	Primer	Primer sequence	Product size (bp)
*vtg*	cRNA FP	TAATACGACTCACTATAGG	
		CATCAAGGAGAAGTTCCTGGCT	401
	cRNA RP	ATTTAGGTGACACTATAGAGGT	
		AGGAGCTTCATGATGGT	
*apo*	cRNA FP	TAATACGACTCACTATAGG	
		GCTTAGTTGAAGTAGTCAGTG	426
	cRNA RP	ATTTAGGTGACACTATAGC	
		CTGACAAGGAGTTAGTTGAGA	
*nasp*	cRNA FP	TAATACGACTCACTATAGG	
		TCAGAGGATTCTTCTGGGACTC	363
	cRNA RP	ATTTAGGTGACACTATAGC	
		GAGGAAACAGCATCTGCTTCA	
*zp2*	cRNA FP	TAATACGACTCACTATAGGGTA	
		CACTTCCTACTACAGTG	372
	cRNA RP	ATTTAGGTGACACTATAGAGATG	
		GTTTCCTGCAGAGGAG	
*vtg*	FP	TGACAGCTAGTTTGGCTATG	149
	RP	AATATCATGGATGGGCCTGA	
*apo*	FP	CACTGGCCAGACCGCTGATAA	155
	RP	GAGGATTGTCACTGCTTGGGA	
*nasp*	FP	CTTCTCAGCTAGCATGGCACAA	133
	RP	GCAGACAGCTCCGTCGATGTTA	
*zp2*	FP	GATGGATGCCCTTACCAGGATG	157
	RP	AGATGGTTTCCTGCAGAGGAG	

Abbreviations: FP, forward primer; RP, reverse primer.

a Primary sequences submitted to GenBank (http://www.ncbi.nih.gov/Genbank).

**Table 2:** Macroarray gene expression results^a^ for female FHM exposed to MeHg-contaminated diets (3.93 ± 0.08 μg MeHg/g dry wt) versus control diets (0.058 ± 0.004 μg MeHg/g dry wt).

Gene name	Fold change	*p*-Value
vitellogenin 1	0.6	0.3113
vitellogenin precursor	0.6	0.4106
glyceraldehyde 3-phosphate dehydrogenase	1.7	0.0263
novel retinal pigment epithelial gene (NORPEG)	2.0	0.3606
piwi protein	2.0	0.5345
phosphoenolpyruvate carboxykinase	2.0	0.2376
heat shock protein 90-beta	2.0	0.2320
L-pipecolic acid oxidase	2.0	0.4516
actin 1	2.0	0.0689
heat shock protein Hsp70	2.0	0.5003
dual specificity phosphatase 13; protein phosphatase	2.0	0.4574
cytosolic branched-chain amino acid aminotransferase	2.0	0.4710
histone H1-0	2.0	0.4968
isocitrate dehydrogenase 2 (NADP+), mitochondrial	2.0	0.4458
G protein pathway suppressor 1; Arabimdopsis FUS6/COP11 homolog	2.1	0.1854
X-box-binding protein 1B	2.1	0.4827
cathepsin D precursor	2.1	0.3563
von Willebrand factor-cleaving protease precursor	2.1	0.4257
pyruvate kinase	2.1	0.5065
A-kinase anchoring protein-associated sperm protein	2.1	0.4567
oxysterol binding protein-like 9 isoform a	2.1	0.2378
mannose binding-like lectin	2.1	0.4514
bile salt export pump	2.2	0.1110
transcription factor JUN-B	2.2	0.3334
glutathione S-transferase 1 (GST-CL1) (GST CLASS-THETA)	2.2	0.4336
ventricular myosin heavy chain	2.3	0.3905
acyl carrier protein, mitochondrial precursor (ACP)	2.3	0.4527
succinyl CoA:3-oxoacid CoA transferase	2.3	0.3305
cell surface glycoprotein HT7 precursor	2.3	0.2830
ubiquitin-like protein SMT3A	2.3	0.4100
protein serine threonine kinase Clk4	2.3	0.4232
cytosolic sulfotransferase	2.3	0.3929
spermatogenesis-preventing substance	2.4	0.3869
apolipoprotein Eb; apolipoprotein E	2.4	0.1711
gonadotropin-regulated long chain acyl-CoA synthetase	2.4	0.4122
GDP-mannose pyrophosphorylase B, isoform 2	2.4	0.4177
ribosomal protein P2	2.4	0.2349
acyl-coenzyme A dehydrogenase, C-4 to C-12 straight chain	2.4	0.3715
adenylate kinase 7	2.4	0.3979
stathmin	2.5	0.3786
cytochrome P450 51	2.5	0.3985
heat shock protein HSP 90-alpha (HSP 86)	2.5	0.4709
acyl-CoA oxidase type 1	2.5	0.3990
ribophorin I	2.5	0.3879
matrix metalloproteinase 9	2.5	0.4384
guanosine monophosphate reductase	2.5	0.4486
beta-ureidopropionase	2.5	0.3735
transposon-derived Buster1 transposase-like protein	2.5	0.3560
creatine kinase, testis isozyme	2.6	0.2586
lanosterol synthase (2,3-oxidosqualene-lanosterol cyclase)	2.6	0.4240
uridine-cytidine kinase 1	2.6	0.4133
CPEB-associated factor Maskin	2.6	0.3647
eukaryotic translation elongation factor 2; polypeptidyl-tRNA translocase	2.6	0.2497
tubulin, alpha 3; tubulin alpha 3	2.6	0.2231
glutathione reductase 1	2.7	0.3697
zinc finger protein 151 (Zinc finger protein Z13)	2.7	0.3620
glucose-6-phosphate-1-dehydrogenase; G6PD	2.7	0.3684
glutamine synthetase	2.7	0.3560
alpha tubulin	2.7	0.2810
eukaryotic translation initiation factor 3, subunit 8	2.8	0.2724
signal peptidase 25 kDA subunit	2.8	0.2700
tubulin beta-1 chain	2.9	0.3540
ependymin precursor (EPD)	2.9	0.3219
S100 calcium-binding protein, beta (neural)	3.0	0.4088
carboxypeptidase B	3.0	0.3561
axonemal dynein heavy chain 7	3.0	0.3940
kainate receptor beta chain precursor	3.0	0.3571
fucosidase, alpha-L-1, tissue	3.1	0.3476
chloride intracellular channel 2	3.1	0.4201
thyroid hormone receptor associated protein complex TRAP230/KIAA0192	3.2	0.2888
angiotensinogen precursor	3.3	0.2102
UDP-glucuronic acid/UDP-N-acetylgalactosamine dual transporter	3.4	0.3009
androgen receptor 1	3.4	0.3362
ubiquitin-conjugating enzyme E2D 2	3.5	0.2676
cyclin A2	3.6	0.1748
asparaginase and ankyrin repeat family member	3.6	0.3353
scavenger receptor cysteine-rich type 1 protein M160	3.8	0.3507
ZP2	9.5	0.1554
ZP3	11.1	0.1349

a Commercial macroarray. Genes names are from GenBank (http://www.ncbi.nih.gov/GenBank) and the European Molecular Biology Laboratory of the European Bioinformatics Institute (EMBL-EBI; http://www.ebi.ac.uk/embl/).

**Table 3:** Macroarray gene expression^a^ results male FHM exposed to MeHg-contaminated diets (3.93 ± 0.08 μg MeHg/g dry wt) versus control diets (0.058 ± 0.004 μg MeHg/g dry wt).

Gene name	Fold change	*p*-Value
phosphoethanolamine methyltransferase	0.2	0.0273
transcription factor JUN-B	0.3	0.3853
fibronectin 1	0.3	0.1483
polyunsaturated fatty acid elongase	0.3	0.3617
serine-pyruvate aminotransferase	0.3	0.2567
fatty acid synthase	0.3	0.1857
glucose-6-phosphatase	0.4	0.0773
intestinal fatty acid binding protein	0.4	0.1941
L-threonine 3-dehydrogenase	0.4	0.3831
annexin VI	0.4	0.2342
superoxide dismutase	0.4	0.0870
transferrin variant D	0.4	0.2122
ubiquitin specific protease 15	0.4	0.2228
cytochrome P450 (2F2)	0.4	0.1455
25-hydroxyvitamin D3 24-hydroxylase	0.5	0.2357
gonadotropin-regulated long chain acyl-CoA synthetase	0.5	0.1703
antithrombin	0.5	0.1259
lanosterol synthase	0.5	0.5225
bile salt export pump	0.5	0.3028
3-hydroxy-3-methylglutaryl-Coenzyme A reductase	0.5	0.3053
cytochrome c oxidase subunit I	0.5	0.2533
4-hydroxyphenylpyruvate dioxygenase	1.9	0.0664
alpha amylase	1.9	0.0895
zinc finger protein 151 (zinc finger protein Z13)	2.0	0.2678
pyruvate kinase	2.0	0.3628
hepatic glucose transporter GLUT2	2.0	0.2632
retinol binding protein 4, plasma	2.0	0.0159
matrix metalloproteinase 9	2.2	0.2291
ventricular myosin heavy chain	2.3	0.2332
biliverdin reductase B [flavin reductase (NADPH)]	2.3	0.4626
kinesin-like protein 2	2.4	0.3089
tubulin beta-1 chain	2.4	0.3005
eukaryotic translation initiation factor 3, subunit 8	2.5	0.1936
cyclin A2	2.5	0.2848
homogentisate 1, 2-dioxygenase	2.6	0.3151
tubulin, alpha 3; tubulin alpha 3	2.8	0.1727
zona pellucida 2 (ZP2)	2.8	0.4347
nucleoside phosphorylase	2.9	0.1578
protein serine threonine kinase Clk4	3.5	0.3601
zona pellucida 3 (ZP3)	14.7	0.3218
vitellogenin precursor	76.3	0.2971
vitellogenin 1	142.8	0.3292

a Commercial macroarray. Genes names from GenBank (http://www.ncbi.nih.gov/GenBank) and the European Molecular Biology Laboratory of the European Bioinformatics Institute (EMBL-EBI; http://www.ebi.ac.uk/embl/).

**Table 4:** qPCR results for all FHM in all treatments measured as molecules per nanogram RNA for each gene product standardized to total RNA.

		Average female		Average male	
Gene locus	Treatment	Molecules/ng (± SE)	Significance (χ^2^, *df*, *p*-value)	Molecules/ng (± SE)	Significance ((χ^2^, *df*, *p*-value)
*vtg*	Control	4.0 × 10^7^ ± 4.8 × 10^6^	(χ^2^ = 9.374, *df* = 2,	6,756 ± 4,284	(χ^2^ = 1.627, *df* = 2,
	Low	9.5 × 10^6^ ± 4.1 × 10^6^	*p* = 0.009*	22,443 ± 18,977	*p* = 0.443
	Medium	8.2 × 10^6^ ± 2.8 × 10^6^		1.4 × 10^6^ ± 1.0 × 10^6^	
*zp2*	Control	2.8 × 10^6^ ± 1.4 × 10^5^	(χ^2^ = 2.279, *df* = 2,	3.0 × 10^5^ ± 74,376	(χ^2^ = 2.920, *df* = 2,
	Low	9.2 × 10^4^ ± 3.0 × 10^4^	*p* = 0.320	4.0 × 10^5^ ± 132,414	*p* = 0.232
	Medium	1.4 × 10^5^ ± 3.7 × 10^4^		1.9 × 10^5^ ± 57,519	
*apo*	Control	2.4 × 10^5^ ± 7.8 × 10^5^	(χ^2^ = 8.861, *df* = 2,	3.4 × 10^6^ ± 7.3 × 10^5^	(χ^2^ = 0.608, *df* = 2,
	Low	5.2 × 10^5^ ± 1.1 × 10^5^	*p* = 0.012*	2.7 × 10^6^ ± 6.3 × 10^5^	*p* = 0.997
	Medium	6.5 × 10^5^ ± 2.2 × 10^5^		3.0 × 10^6^ ± 9.4 × 10^5^	
*nasp*	Control	527± 267	(χ^2^ = 6.151, *df* = 2,	540 ± 178	(χ^2^ = 0.106, *df* = 2,
	Low	14,711 ± 9,459	*p* = 0.046*	3,079 ± 227	*p* = 0.948
	Medium	21,351 ± 14,738		1,098 ± 494	

* Significant at *p* = 0.05
