# Supplementary material for: Gene Expression Changes Related to Endocrine Function and Decline in Reproduction in Fathead Minnow (Pimephales promelas) after Dietary Methylmercury Exposure
Source: Environ Health Perspect. 2006 May 30;114(9):1337–43. doi: 10.1289/ehp.8786 (PMC1570078; doi:10.1289/ehp.8786)
Supplement: Supplemental Figures and Tables [file ehp0114-001337s1.pdf]

EcoArray Gene Number	Top hit definition (blast X)
EA-FHM-001	fibronectin 1
EA-FHM-002	beta-ureidopropionase
EA-FHM-003	L-pipecolic acid oxidase
EA-FHM-004	Vitronectin precursor (S-protein) (Glycoprotein 66)
EA-FHM-005	isocitrate dehydrogenase 2 (NADP+), mitochondrial
EA-FHM-006	X-box-binding protein 1B
EA-FHM-007	ribosomal protein S8
EA-FHM-008	apolipoprotein Eb; apolipoprotein E
EA-FHM-009	ribosomal protein S17
EA-FHM-010	actin 1
EA-FHM-011	GDP-mannose pyrophosphorylase B, isoform 2;
EA-FHM-012	carboxypeptidase B
EA-FHM-013	antithrombin
EA-FHM-014	ribosomal protein P2
EA-FHM-015	transmembrane 4 superfamily member protein
EA-FHM-016	fatty acid binding protein 10, liver basic
EA-FHM-017	sarcosine dehydrogenase
EA-FHM-018	Fatty acid synthase
EA-FHM-019	cytochrome-c oxidase (EC 1.9.3.1) chain II
EA-FHM-020	transposon-derived Buster1 transposase-like protein
EA-FHM-021	alpha tubulin
EA-FHM-022	3-hydroxy-3-methylglutaryl-Coenzyme A reductase
EA-FHM-023	pre-alpha-inhibitor, heavy chain 3
EA-FHM-024	aldose 1-epimerase
EA-FHM-025	scavenger receptor cysteine-rich type 1 protein M160
EA-FHM-026	cell surface glycoprotein HT7 precursor
EA-FHM-027	protein serine threonine kinase Clk4
EA-FHM-028	von Willebrand factor-cleaving protease precursor
EA-FHM-029	Microsomal glutathione S-transferase 1 (Microsomal GST-1)
EA-FHM-030	aquaporin X2
EA-FHM-031	sulfite oxidase
EA-FHM-032	apolipoprotein B
EA-FHM-033	NADH dehydrogenase subunit 2
EA-FHM-034	ATP synthase 6
EA-FHM-035	cytosolic sulfotransferase
EA-FHM-036	phosphoenolpyruvate carboxykinase
EA-FHM-037	complement component C9
EA-FHM-038	heat shock protein 90-beta
EA-FHM-039	acyl-CoA oxidase type 1
EA-FHM-040	cytochrome P4501A
EA-FHM-041	thyroid hormone receptor associated protein complex component
EA-FHM-042	glucose-6-phosphate-1-dehydrogenase; G6PD
EA-FHM-043	matrix metalloproteinase 9
EA-FHM-044	serine-pyruvate aminotransferase
EA-FHM-045	25-hydroxyvitamin D3 24-hydroxylase
EA-FHM-046	serine hydroxymethyltransferase 1 (soluble)
EA-FHM-047	polyunsaturated fatty acid elongase

EA-FHM-048	bile salt export pump
EA-FHM-049	upregulated by 1,25-dihydroxyvitamin D-3
EA-FHM-050	heat shock cognate 70 kDa protein
EA-FHM-051 1)	Polyadenylate-binding protein 1 (Poly(A)-binding protein 1) (PABP 4.16E-34
EA-FHM-052	oxysterol binding protein-like 9 isoform a
EA-FHM-053	lysosomal cofactor/neurotrophic factor prosaposin
EA-FHM-054	UDP-glucose dehydrogenase
EA-FHM-055	Arabidopsis FUS6/COP11 homolog
EA-FHM-056	hepatocyte nuclear factor 4 alpha
EA-FHM-057	mammary apoptosis inducing factor
EA-FHM-058	calcium activated chloride channel 4
EA-FHM-059	CYP2J4
EA-FHM-060	cytochrome P450 (2F2)
EA-FHM-061	hydroxy-delta-5-steroid dehydrogenase
EA-FHM-062	L-threonine 3-dehydrogenase
EA-FHM-063	selenoprotein Pa
EA-FHM-064	4-hydroxyphenylpyruvate dioxygenase
EA-FHM-065	phenylalanine hydroxylase
EA-FHM-066	receptor for activated protein kinase C
EA-FHM-067	carnitine palmitoyltransferase I
EA-FHM-068	glucose 6-phosphate translocase
EA-FHM-069	renal organic cation transporter
EA-FHM-070	fibrinogen alpha
EA-FHM-071	delta-6 fatty acyl desaturase
EA-FHM-072	eukaryotic translation elongation factor 2
EA-FHM-073	inter-alpha trypsin inhibitor, heavy chain 2
EA-FHM-074	heat shock protein 47
EA-FHM-075	plasma hyaluronan-binding protein precursor
EA-FHM-076	hemicentin
EA-FHM-077	alcohol dehydrogenase
EA-FHM-078	annexin VI
EA-FHM-079	catalase
EA-FHM-080	nucleoside phosphorylase
EA-FHM-081	UDP-glucose pyrophosphorylase
EA-FHM-082	TRANSCRIPTION FACTOR JUN-B
EA-FHM-083	ceruloplasmin (ferroxidase)
EA-FHM-084	cyclin G
EA-FHM-085	R3H domain (binds single-stranded nucleic acids) containing
EA-FHM-086	signal peptidase 25 kDA subunit
EA-FHM-087	ZP3
EA-FHM-088	ZP2
EA-FHM-089	ventricular myosin heavy chain
EA-FHM-090	pyruvate kinase
EA-FHM-091	ATP synthase beta-subunit
EA-FHM-092	NADH dehydrogenase subunit 1
EA-FHM-093	spermatogenesis-preventing substance
EA-FHM-094	A-kinase anchoring protein-associated sperm protein
EA-FHM-095	CPEB-associated factor Maskin
EA-FHM-096	histone H1-0
EA-FHM-097	Superoxide dismutase
EA-FHM-098	ferritin H-1
EA-FHM-099	intestinal fatty acid binding protein

EA-FHM-100	3-oxoacid CoA transferase precursor;
EA-FHM-101	transferrin variant D
EA-FHM-102	Protein C
EA-FHM-103	UDP-glucuronic acid/UDP-N-acetylgalactosamine dual transporter
EA-FHM-104	cytochrome c oxidase subunit I
EA-FHM-105	elongation factor 1-alpha; EF-1-alpha
EA-FHM-106	retinol binding protein 2, cellular
EA-FHM-107	fucosidase, alpha-L- 1, tissue
EA-FHM-108	glutathione peroxidase
EA-FHM-109	Acrosin-trypsin inhibitor II precursor (Serine protease inhibitor
Kazal-type 2)	5.94E-12
EA-FHM-110	serine proteinase inhibitor CP9 - common carp
EA-FHM-111	cadherin 1, epithelial
EA-FHM-112	ribosomal protein L4
EA-FHM-113	vitellogenin 1
EA-FHM-114	(NADH-ubiquinone oxidoreductase 9.6 kDa subunit) (CI-SDAP)
EA-FHM-115	guanosine monophosphate reductase
EA-FHM-116	cytochrome P450, family 46 (cholesterol 24-hydroxylase)
EA-FHM-117	vitellogenin precursor
EA-FHM-118	retinoic acid induced 14; novel retinal pigment epithelial gene;
EA-FHM-119	phosphoethanolamine methyltransferase
EA-FHM-120	cathepsin D precursor
EA-FHM-121	complement C3-S
EA-FHM-122	uridine-cytidine kinase 1
EA-FHM-123	ribosomal protein S6
EA-FHM-124	glutathione S-transferase 1(GST-CL1) (GST CLASS-THETA)
EA-FHM-125	alpha-2-macroglobulin-2
EA-FHM-126	ribophorin I
EA-FHM-127	insulin receptor substrate-1-like protein
EA-FHM-128	mannose binding-like lectin
EA-FHM-129	28kDa-2 apolipoprotein
EA-FHM-130	ribosomal protein S4
EA-FHM-131	lanosterol synthase (2,3-oxidosqualene-lanosterol cyclase)
EA-FHM-132	ribosomal protein L21
EA-FHM-133	warm-temperature-acclimation-related-65 kDa-protein-like-protein
EA-FHM-134	complement C3-H1
EA-FHM-135	acyl-Coenzyme A dehydrogenase, C-4 to C-12 straight chain
EA-FHM-136	complement B/C2-A2
EA-FHM-137	solute carrier family 25 alpha, member 5
EA-FHM-138	ubiquitin-conjugating enzyme E2D 2
EA-FHM-139	cytochrome P450 51
EA-FHM-140	Amiloride binding protein 1
EA-FHM-141	alpha-1-microglobulin/inter-alpha-trypsin inhibitor precursor
EA-FHM-142	glyceraldehyde 3-phosphate dehydrogenase
EA-FHM-143	Heat shock protein HSP 90-alpha (HSP 86)
EA-FHM-144	APOLIPOPROTEIN A-IV PRECURSOR (APO-AIV)
EA-FHM-145	gonadotropin-regulated long chain acyl-CoA synthetase
EA-FHM-146	3-hydroxyanthranilate 3,4-dioxygenase
EA-FHM-147	chloride intracellular channel 2
EA-FHM-148	S100 calcium-binding protein, beta (neural)
EA-FHM-149	androgen receptor 1
EA-FHM-150	glutamine synthetase
EA-FHM-151	kainate receptor beta chain precursor

EA-FHM-152	EPENDYMIN PRECURSOR (EPD)
EA-FHM-153	Glucose-6-phosphatase (G6Pase) (G-6-Pase)
EA-FHM-154	aldolase B
EA-FHM-155	alpha-2-macroglobulin-1
EA-FHM-156	6-phosphofructo-2-kinase/fructose-2,6-bisphospha tase
EA-FHM-157	14kDa apolipoprotein
EA-FHM-158	angiotensinogen precursor
EA-FHM-159	cyclin A2
EA-FHM-160	Asparaginase and ankyrin repeat family member
EA-FHM-161	Zinc finger protein 151 (Zinc finger protein Z13)
EA-FHM-162	glutathione reductase 1
EA-FHM-163	axonemal dynein heavy chain 7
EA-FHM-164	TUBULIN BETA-1 CHAIN
EA-FHM-165	ubiquitin specific protease 15
EA-FHM-166	stathmin
EA-FHM-167	Ubiquitin-like protein SMT3A
EA-FHM-168	piwi protein
EA-FHM-169	kinesin-like protein 2
EA-FHM-170	heat shock protein Hsp70
EA-FHM-171	CREATINE KINASE, TESTIS ISOZYME
EA-FHM-172	vitamin-D binding protein precursor
EA-FHM-173	androgen-binding protein precursor
EA-FHM-174	homogentisate 1, 2-dioxygenase
EA-FHM-175	tubulin, alpha 3; tubulin alpha 3
EA-FHM-176	eukaryotic translation initiation factor 3, subunit 8
EA-FHM-177	adenylate kinase 7
EA-FHM-178	retinol binding protein 4, plasma; retinol binding protein
EA-FHM-179	cytosolic branched-chain amino acid aminotransferase
EA-FHM-180	dual specificity phosphatase 13; protein phosphatase
EA-FHM-181	histone H2A.F/Z variant, isoform 1; purine-rich binding element
protein B	4.14E-25
EA-FHM-182	biliverdin reductase B (flavin reductase (NADPH))
EA-FHM-183	Fructose-1,6-bisphosphatase (FBPase)
EA-FHM-184	dihydropyrimidinase
EA-FHM-185	phospholipid hydroperoxide glutathione peroxidase
EA-FHM-186	hepatic glucose transporter GLUT2
EA-FHM-187	Discoidin domain receptor 2 precursor (Receptor protein-tyrosine
kinase TKT)	1.68E-40
EA-FHM-188	alpha amylase
	A. thaliana - CAB
	A. thaliana - RCA
	A. thaliana - rcbL
	Human COT-1
